# Global Survey and Genome Exploration of Bacteriophages Infecting the Lactic Acid Bacterium *Streptococcus thermophilus*

**DOI:** 10.3389/fmicb.2017.01754

**Published:** 2017-09-12

**Authors:** Brian McDonnell, Jennifer Mahony, Laurens Hanemaaijer, Horst Neve, Jean-Paul Noben, Gabriele A. Lugli, Marco Ventura, Thijs R. Kouwen, Douwe van Sinderen

**Affiliations:** ^1^School of Microbiology, College of Science, Engineering and Food Science, University College Cork Cork, Ireland; ^2^APC Microbiome Institute, University College Cork Cork, Ireland; ^3^DSM Biotechnology Centre Delft, Netherlands; ^4^Department of Microbiology and Biotechnology, Max Rubner-Institut Kiel, Germany; ^5^Biomedical Research Institute, Hasselt University Diepenbeek, Belgium; ^6^Laboratory of Probiogenomics, Department of Life Sciences, University of Parma Parma, Italy

**Keywords:** panvirome, methyltransferase, transcriptional regulator, structural proteome, antireceptor

## Abstract

Despite the persistent and costly problem caused by (bacterio)phage predation of *Streptococcus thermophilus* in dairy plants, DNA sequence information relating to these phages remains limited. Genome sequencing is necessary to better understand the diversity and proliferative strategies of virulent phages. In this report, whole genome sequences of 40 distinct bacteriophages infecting *S. thermophilus* were analyzed for general characteristics, genomic structure and novel features. The bacteriophage genomes display a high degree of conservation within defined groupings, particularly across the structural modules. Supporting this observation, four novel members of a recently discovered third group of *S. thermophilus* phages (termed the 5093 group) were found to be conserved relative to both phage 5093 and to each other. Replication modules of *S. thermophilus* phages generally fall within two main groups, while such phage genomes typically encode one putative transcriptional regulator. Such features are indicative of widespread functional synteny across genetically distinct phage groups. Phage genomes also display nucleotide divergence between groups, and between individual phages of the same group (within replication modules and at the 3′ end of the lysis module)—through various insertions and/or deletions. A previously described multiplex PCR phage detection system was updated to reflect current knowledge on *S. thermophilus* phages. Furthermore, the structural protein complement as well as the antireceptor (responsible for the initial attachment of the phage to the host cell) of a representative of the 5093 group was defined. Our data more than triples the currently available genomic information on *S. thermophilus* phages, being of significant value to the dairy industry, where genetic knowledge of lytic phages is crucial for phage detection and monitoring purposes. In particular, the updated PCR detection methodology for *S. thermophilus* phages is highly useful in monitoring particular phage group(s) present in a given whey sample. Studies of this nature therefore not only provide information on the prevalence and associated threat of known *S. thermophilus* phages, but may also uncover newly emerging and genomically distinct phages infecting this dairy starter bacterium.

## Introduction

The problem of phage predation of *Streptococcus thermophilus* in the dairy industry has been well described (Caldwell et al., 1996; Bruttin et al., 1997; Quiberoni et al., 2006; Garneau and Moineau, 2011), though the precise impact on the fermentation process can only be estimated due to logistical limitations and commercial sensitivities. A crucial first step in tackling this problem is the availability of comprehensive genetic information on both host and virus. The advent of the “genomics age,” as facilitated through advanced sequencing technologies (reviewed in the context of dairy starter selection by Kelleher et al., 2015), has enabled the accumulation of genetic data on many (groups of) phages, not least those infecting the most commonly employed dairy starter bacterium, *Lactococcus lactis* (Fortier et al., 2006; Dupuis and Moineau, 2010; Murphy et al., 2014; Mahony et al., 2015). Despite having access to full genomic data sets of both *S. thermophilus* hosts (Goh et al., 2011; Sun et al., 2011; Wu et al., 2014) and their infecting phages (Guglielmotti et al., 2009b; Mills et al., 2011; Ali et al., 2014), the amount of publicly released genetic data relating to lytic phages of *S. thermophilus* remains rather limited.

To date, the complete genomes of 20 phages infecting *S. thermophilus* have been published: O1205 (Stanley et al., 1997), Sfi19 and Sfi21 (Desiere et al., 1998), DT1 (Tremblay and Moineau, 1999), Sfi11 (Lucchini et al., 1999), 7201 (Stanley et al., 2000), 2972 (Levesque et al., 2005), 858 (Deveau et al., 2008), ALQ13.2 and Abc2 (Guglielmotti et al., 2009b), 5093 (Mills et al., 2011), TP-J34L and TP-778L (Ali et al., 2014), 9871, 9872, 9873, and 9874 (McDonnell et al., 2016), and (very recently) CHPC577, CHPC926, and CHPC1151 (Szymczak et al., 2016). Their availability revealed that *S. thermophilus* phage genomes possess a modular structure, while it also allowed an analysis of their evolution and relatedness (Lucchini et al., 1999; Proux et al., 2002), thereby providing insights into some unusual genetic lineages (Mills et al., 2011; McDonnell et al., 2016; Szymczak et al., 2016; discussed further below). It has, furthermore, improved our understanding on how phage-host interactions cause iterative genomic changes. For example, it is now known that the *S. thermophilus* CRISPR (Clustered Regularly Interspaced Short Palindromic Repeats)-Cas phage-resistance system relies on the acquisition of short genomic regions from the infecting phage (Hols et al., 2005; Barrangou et al., 2007; Deveau et al., 2008), which in turn is counteracted by the accumulation of point mutations in the phage genome (Deveau et al., 2008), leading to iterative genomic alterations.

The emergence of the 5093 group (Mills et al., 2011) was a significant discovery in terms of expanding the biological diversity of *S. thermophilus* phages. While previously described phages (and their genomes) could be assigned to either the so-called *cos*-containing or *pac*-containing groups (Le Marrec et al., 1997), the genome of phage 5093 instead displays a striking sequence similarity to phages that infect non-dairy streptococcal species. Furthermore, the apparent absence of an antireceptor (the phage tail tip component involved in the initial adsorption to the host cell) suggests that this phage is fundamentally different in its adsorption mechanism compared to previously identified *S. thermophilus* phages (Duplessis and Moineau, 2001). The expanding genetic diversity of *S. thermophilus* phages is further highlighted by the recent discovery of the so-called 987 group phages (McDonnell et al., 2016). Members of this novel phage group appear to have evolved through a genetic exchange event between an *S. thermophilus* phage and an unknown member of the P335 group of *L. lactis* phages (Labrie et al., 2008). Recently, the genome sequences of further phages, exhibiting similarity to 5093, as well as two additional phages exhibiting similarity to P335 group *L. lactis* phages, have been determined (Szymczak et al., 2016).

It is clear that there is a need to expand the amount of genetic information available in this field, particularly in light of the emergence of genetically divergent phage groups described above. Here, we present whole genome sequences of 40 phages infecting *S. thermophilus*. This sequencing project triples the number of available full genomic sequences of *S. thermophilus* phages. The sequenced genomes were assessed for general structural characteristics as well as modular configurations. A multiplex PCR detection and classification system described previously was updated to include recently discovered phage groups. Novel features which may confer a selective advantage to the phage in terms of infection or persistence capability are indicated. In addition, the structural protein complement and receptor-binding protein (termed the “antireceptor”) of a representative 5093 group phage was defined, significantly updating the genomic annotation of this phage group.

## Materials and methods

### Phage propagation, enumeration, and storage

*Streptococcus thermophilus* strains were routinely grown from single colonies or from 10% Reconstituted Skimmed Milk (RSM) stocks overnight at 42°C in M17 Broth (Oxoid, Hampshire, U.K.) supplemented with 0.5% lactose (Sigma-Aldrich, St. Louis, MO, U.S.A.; LM17). *L. lactis* NZ9000 was maintained at 30°C in M17 broth containing 0.5% glucose (Sigma-Aldrich; GM17), while NZ9000 derivatives containing pNZ8048-based constructs were maintained in GM17 with the addition of 5 μg/ml chloramphenicol (Sigma-Aldrich; GM17 + Cm5). Phage enumeration was performed using a standard method (Lillehaug, 1997), for which LM17 broth was supplemented with 0.25% glycine (Sigma-Aldrich), 10 mM CaCl_2_ (Sigma-Aldrich) and either 10 g/L (solid agar base) or 4 g/L (semi-solid overlay) Technical Agar (Merck, Darmstadt, Germany). Whey samples from dairy plants producing fermented milk products were obtained and analyzed for the presence of phages against *S. thermophilus* using spot and plaque assay methods as described above. Single plaque isolates were then propagated as follows: 10 ml LM17 broth was inoculated with 100 μl of the appropriate host strain and grown at 42°C for 1.5–2.0 h. Using a sterile pipette tip, a single, well defined plaque was added to the growing culture, thoroughly mixed and incubated for a further 2–4 h or overnight. The lysed culture was centrifuged and the supernatant filtered (0.45 μm). The filtered supernatant was stored at 4°C and used as stock for subsequent assays. This process was repeated at least twice prior to DNA sample preparation and subsequent genome sequencing.

### Phage purification

Individual phages (Table 1) were propagated as described above in a 2 L volume before the addition of (poly)ethylene glycol (Sigma-Aldrich) to a final volume of 10% (w/v) and NaCl (Sigma-Aldrich) to a final concentration of 0.5 M. The mixtures were incubated at 4°C for at least 6 h to encourage precipitation before centrifugation at 17,700 × *g* for 15 min (to concentrate) and resuspension in 5 ml TBT Buffer (100 mM NaCl, 100 mM Tris-HCl (pH 7), 10 mM MgCl_2_, 20 mM CaCl_2_; Sigma-Aldrich). The suspension was extracted at least twice using an equal volume of chloroform (Fisher Scientific, Waltham, MA, U.S.A.) and phages were purified by a discontinuous (3 M/5 M) cesium chloride (Sigma-Aldrich) gradient centrifugation at 76,000 × *g* for 2.5 h. Translucent blue bands visible at the interface of the gradient after centrifugation were carefully removed using a syringe and dialyzed against 50 ml TBT overnight at 4°C. Phage preparations were stored at 4°C until required for electron microscopy and DNA extraction.

**Table 1:** Characteristics of bacteriophage genomes sequenced during this study.

**Phage**	**Host**	**Country of origin**	**Technology**	**Genome size (bp)**	**#ORFs**	**Coding (%)**	**GC content (%)**	**Group**	**Replication**	**Ltr**	**Notable features**	**Genbank accession #**
0091	ST67009	Germany	454	35653	44	95.1	39.2	*Cos*	7201-like (II)	Ltrb	RecT, RusA, MTase (x2), HNH (x2)	KY705251
0092	ST67009	Germany	454	34580	50	94.9	38.4	5093 group	7201-like (II)	ArpU	RecT, RusA, MTase (x2), HNH	KY705252
0093	ST67009	France	454	34932	51	95.4	38.6	5093 group	7201-like (II)	ArpU	RecT, RusA, MTase (x2), HNH	KY705253
0094	ST67009	France	454	33335	47	94.6	38.6	5093 group	7201-like (II)	ArpU	RecT, RusA, MTase (x2), HNH	KY705254
0095	ST67009	Turkey	454	35966	55	92.7	38.1	5093 group	7201-like (II)	ArpU	RecT, RusA, MTase (x2)	KY705255
3681	ST67368	Italy	454	36168	46	92.8	38.4	*Cos*	Sfi21-like (I)	Ltrb	HNH	KY705256
3684	ST67368	Italy	454	35444	45	91.9	38.9	*Cos*	Sfi21-like (I)	Ltrb	HNH	KY705257
4761	ST64476	France	454	**38139**	55	93.3	38.9	*Pac*	7201-like (II)	Ltrb	ERF	KY705258
5641	ST66564	France	454	37235	52	93.5	38.9	*Cos*	7201-like (II)	Ltrb	RecT, Nuclease, RusA, HNH (x2)	KY705259
5651	ST66565	Colombia	454	34292	43	91.4	39.1	*Cos*	Sfi21-like (I)	Ltrb	HNH	KY705260
5652	ST66565	U.K.	454	33448	43	92.8	39.3	*Cos*	Sfi21-like (I)	Ltrb	HNH	KY705261
7132	ST64713	Austria	454	35843	45	92.8	39.0	*Cos*	Sfi21-like (I)	Ltrb	HNH	KY705262
7133	ST64713	Spain	454	**33284**	42	93.8	38.8	*Cos*	Sfi21-like (I)	Ltrb	HNH	KY705263
7134	ST64713	U.K.	454	34019	45	93.6	38.9	*Cos*	Sfi21-like (I)	Ltrb	HNH	KY705264
7151	ST64715	Japan	454	35025	47	92.6	38.8	*Cos*	Sfi21-like (I)	Ltrb	HNH	KY705265
7152	ST64715	U.K.	454	35663	46	92.7	38.9	*Cos*	Sfi21-like (I)	Ltrb	HNH	KY705266
7154	ST64715	Colombia	454	34507	42	93.2	38.8	*Cos*	Sfi21-like (I)	Ltrb	HNH	KY705267
7571	ST68757	France	454	34907	49	92.5	39.4	*Pac*	Sfi21-like (I)	ArpU		KY705268
7572	ST68757	The Netherlands	454	33410	43	92.3	39.6	*Pac*	Sfi21-like (I)	ArpU		KY705269
7573	ST68757	Turkey	454	37715	49	92.7	38.7	*Cos*	Sfi21-like (I)	Ltrb	HNH	KY705270
7574	ST68757	Morocco	MiSeq	36298	50	90.3	38.8	*Cos*	7201-like (II)	Ltrb	IS, RecT, Nuclease, RusA, HNH	KY705271
7601	ST69760	Germany	454	35395	51	94.3	38.5	*Cos*	Sfi21-like (I)	Ltrb	HNH	KY705272
7602	ST69760	USA	454	37202	51	94.7	38.7	*Cos*	Sfi21-like (I)	Ltrb	HNH	KY705273
7631	ST69763	France	454	36164	48	92.7	38.9	*Cos*	Sfi21-like (I)	Ltrb	HNH	KY705274
7632	ST69763	Germany	454	35988	48	92.9	39.1	*Cos*	Sfi21-like (I)	Ltrb	Transposase (x2), HNH	KY705275
7633	ST69763	Germany	454	35921	47	93.1	38.9	*Cos*	Sfi21-like (I)	Ltrb	HNH	KY705276
7951	ST47795	France	454	34123	47	93.5	39.8	*Pac*	Non-I/Non-II	ArpU	RecT, Nuclease, RusA	KY705277
7952	ST47795	Turkey	454	37594	48	93.7	39.0	*Pac*	Sfi21-like (I)	ArpU	Transposase (x2)	KY705278
7953	ST47795	Turkey	454	35786	43	93.0	39.1	*Pac*	Sfi21-like (I)	ArpU		KY705279
7954	ST47795	Turkey	454	35688	46	94.3	38.9	*Pac*	Sfi21-like (I)	ArpU		KY705280
7955	ST47795	The Netherlands	MiSeq	36931	50	93.5	38.9	*Pac*	Non-I/Non-II	ArpU	RecT, Nuclease, RusA	KY705281
8921	ST64892	Japan	454	34516	46	92.6	39.0	*Cos*	Sfi21-like (I)	Ltrb	HNH	KY705282
8922	ST64892	U.K.	454	34509	43	93.6	38.8	*Cos*	Sfi21-like (I)	Ltrb	HNH	KY705283
9851	ST64985	France	MiSeq	35835	48	94.0	39.1	*Cos*	Sfi21-like (I)	Ltrb	HNH	KY705284
9852	ST64985	France	MiSeq	34638	44	93.3	40.0	*Pac*	Sfi21-like (I)	ArpU		KY705285
9853	ST64985	Austria	MiSeq	34469	45	93.3	39.7	*Pac*	Sfi21-like (I)	ArpU		KY705286
9854	ST64985	The Netherlands	MiSeq	36979	48	93.8	38.9	*Cos*	Sfi21-like (I)	Ltrb	HNH	KY705287
9901	ST62990	Germany	MiSeq	35211	47	94.1	38.9	*Cos*	7201-like (II)	Ltrb	RusA, HNH	KY705288
9902	ST62990	France	454	35535	51	94.9	38.9	*Cos*	7201-like (II)	Ltrb	MTase, HNH (x2)	KY705289
9903	ST62990	Czech Republic	MiSeq	35256	50	93.7	38.7	*Cos*	7201-like (II)	Ltrb	MTase, RusA, HNH	KY705290

*The longest and shortest genomes sequenced are highlighted in bold*.

### DNA preparation and restriction profile analyses

Phage DNA was prepared using existing methods (Sambrook et al., 1989; Moineau et al., 1994), and as described previously (McDonnell et al., 2016). At least 5 μg DNA was extracted using this method and quantified using a NanoDrop 2000 (Thermo Scientific). Total genomic DNA was qualitatively analyzed by agarose (1%; Sigma-Aldrich) gel electrophoresis, on which the samples were separated at 100 V for at least 20 min.

Unique phages were distinguished (prior to DNA sequencing) by restriction profile analysis using EcoRV or HaeIII FastDigest (Thermo Scientific) enzymes, whereby a reaction mixture containing 8 μl phage DNA, 1 μl enzyme, 2 μl 10X FastDigest Buffer and 9 μl sterile distilled water (sdH_2_O) was incubated at 37°C for 17 min prior to electrophoretic examination as described above.

### Multiplex PCR detection of phages

PCR multiplex detection of *S. thermophilus* phage groups was performed using phage DNA as template and is a modification of a previously described method (Quiberoni et al., 2006). Two primer sets, each with a target in the 987 and 5093 group phages, were added to the PCR reaction in addition to the two previously described primer sets (targeting *cos*- and *pac*-containing phages; Table 2). PCR reactions were conducted in a total volume of 25 μl using Taq polymerase (Qiagen, Hilden, Germany). Reaction conditions were as follows: initial denaturation of 95°C for 2 min, 30 cycles of 95°C for 15 s, 55°C for 30 s, and 72°C for 1 min, with a final extension of 72°C for 10 min.

**Table 2:** PCR primers applied during this study.

**Primer name**	**Sequence (5′–3′)**	**Sources**
**PHAGE DETECTION**		
CosF	GGTTCACGTGTTTATGAAAAATGG	Quiberoni et al., 2006
CosR	AGCAGAATCAGCAAGCAAGCTGTT	Quiberoni et al., 2006
PacF	GAAGCTATGCGTATGCAAGT	Quiberoni et al., 2006
PacR	TTAGGGATAAGAGTCAAGTG	Quiberoni et al., 2006
987F	CTAAGCGTTTGCCACTGTCAG	This study
987R	GCTGCCGCTTGTTTGAAAAC	This study
5093F	CTGGCTCTTGGTGGTCTTGC	This study
5093R	GCGGCAACCATCTTAGACCAG	This study
**RBP_0095_ CLONING**		
RBP0095F	AGCAGCCCATGGAACACCATCACCATCACCATT CTTCTGGTCAAACAGAAGCAGAGGGA	This study
RBP0095R	AGCAGCCTGCAGGCACACTCAATGATCGTGTTT	This study

### DNA sequencing and *in silico* analysis

Phage DNA samples exhibiting unique restriction pattern profiles were sequenced using one of two methods, as follows: (i) random shotgun sequencing using pyrosequencing technology was performed (Macrogen, Inc., Geumcheon-gu, Seoul, South Korea) using a 454 FLX instrument yielding at least 65-fold coverage for each genome, with the exception of phage 7632, which yielded 17-fold coverage. Individual sequence files were assembled using GSassembler (454 Lifesciences, Branford, CT, U.S.A.), generating a consensus sequence for each phage; (ii) The remaining genomes were determined by GenProbio srl (University of Parma, Italy) using a MiSeq sequencer (Illumina, USA). Genomic libraries were constructed employing the TruSeq Nano DNA LT Kit (Illumina) and using 200 ng of genomic DNA, which was fragmented with a Bioruptor NGS ultrasonicator (Diagenode, USA) followed by size evaluation using Tape Station 2200 (Agilent Technologies). Library samples were loaded into a Flow Cell V3 600 cycles (Illumina) according to the technical support guide. The generated paired-end reads (2 × 251 bp read lengths) were depleted of adapter sequences, quality filtered and assembled through the MIRA software program (version 4.0.2; Chevreux et al., 2004) and the MEGAnnotator pipeline (which was also used to perform the initial gene annotation; Lugli et al., 2016), where a coverage level of at least 158-fold was achieved. For all genomes, remaining gaps were closed and quality improvement of the genomes was carried out (particularly across homopolymeric tracts) by PCR and additional Sanger sequencing (performed by Eurofins MWG, Ebersberg, Germany). ORFs on each genome, representing putative protein products, were predicted using a Heuristic approach (Genemark; Besemer and Borodovsky, 1999) and annotated using the Basic Local Alignment Search Tool (Altschul et al., 1990), as well as Pfam (Finn et al., 2014), and HHpred (Soding et al., 2005). Amino acid identity between phage-encoded proteins was determined using BLASTP. Percent identity and divergence tables (Supplementary Figure S1) were constructed using Megalign [DNASTAR; version 7.1.0 (44)]. Phage VR2 region genetic relatedness trees were generated using the MEGA program (Tamura et al., 2013). Pan-genome analysis of a total of 57 *S. thermophilus* phage genomes was performed using PGAP v1.0 (Zhao et al., 2012), which employs the Heaps law pan-genome model (Tettelin et al., 2005). The pan-genome profile was built by firstly organizing the ORF content of each genome into functional gene clusters using the Gene Family method (Zhao et al., 2012).

### Electron microscopic analysis

Cesium chloride purified phage samples were prepared as outlined above and subjected to electron microscopic analysis as described previously (Vegge et al., 2005; Casey et al., 2015).

### Structural protein identification

Methanol-chloroform extraction of phage proteins, SDS-PAGE visualization and preparation of phage structural protein samples for mass spectrometry were performed as described previously (Casey et al., 2015). Electrospray ionization-tandem mass spectrometry (ESI-MS/MS) was performed as previously described (Ceyssens et al., 2008; Vanheel et al., 2012). For each peptide identified, coverage levels of two unique amino acid strings, or at least 5% of the total protein, were used as cut-off values when identifying gene products as components of the viral particle (Cornelissen et al., 2012).

### Protein purification and adsorption inhibition assay

Proteins were purified using a method adapted from a previously described methodology (Collins et al., 2013). Firstly, *ORF18*_0095_ (encoding RBP_0095_), and its attached 6xHis N-terminal-specifying purification tag and appropriate restriction enzyme recognition sequence was PCR amplified (using the primers described in Table 2) employing Phusion polymerase (New England Biolabs, Ipswich, MA, U.S.A.) and cloned behind the Nisin-inducible promoter of lactococcal plasmid pNZ8048 (Kuipers et al., 1998). Plasmid DNA was dialysed against sdH_2_O for 10 min and transformed into electrocompetent *L. lactis* NZ9000 cells (Kuipers et al., 1998). Plasmid DNA was then extracted using the GeneJet Plasmid Miniprep Kit (Thermo Scientific) and subjected to Sanger sequencing (as above) to confirm the expected sequence of the recombinant plasmid, which was termed pNZ8048+*RBP*_0095_. NZ9000 containing plasmid pNZ8048+*RBP*_0095_ were grown to an OD_600nm_ of 0.2 before nisin induction [10 ng/ml; using Nisaplin (Danisco, Copenhagen, Denmark)], cell lysis, and sonication as previously described (Collins et al., 2013). Target protein purification was then performed using a Ni-nitrilotriacetic acid (NTA) agarose (Qiagen) column (Bio-Rad, Hercules, CA, U.S.A.) as previously described (Collins et al., 2013). Protein fractions were eluted using varying concentrations of imidazole buffer (as per manufacturer's instructions) and separated on a 12.5% SDS-PAGE gel at 160 V for 80 min. Fractions containing bands of the correct size with minimal contamination were dialysed against 100 ml protein buffer (as above) three times for 40 min to remove remaining imidazole. Dialysed fractions were stored at 4°C for use in subsequent assays.

Adsorption inhibition assays were performed using a method adapted from two previously reported methods (Garvey et al., 1996; Collins et al., 2013). Briefly, the *S. thermophilus* strain to be tested was grown to at least OD_600nm_ 0.5 (yet not above 0.54) and resuspended in 225 μl $\frac{1}{4}$ strength Ringer's solution (Merck). 50 μl of varying concentrations of purified antireceptor protein (or protein buffer control) was added to cells (or $\frac{1}{4}$ strength Ringer's solution control) and incubated for 1 h at 42°C. Phage lysates (225 μl), diluted in $\frac{1}{4}$ strength Ringer's solution to a concentration of approximately 1 × 10^5^ pfu/ml were added to the cells or control and the mixture was incubated at 42°C for a further 12 min before centrifugation at 20,000 × *g* for 3 min to pellet bound phages with the cells. Remaining phages were quantified by plaque assay (as described above). Adsorption to wild-type and antireceptor-incubated cells was calculated as follows: (control phage titer − sample phage titer)/(control phage titer) × 100. Adsorption inhibition, expressed as a percentage of phage adsorption to wild type cells, was calculated as follows: (% adsorption on WT − % adsorption on incubated cells)/(% adsorption on WT) × 100.

## Results and discussion

### Isolation of phages, genotype grouping, selection for sequencing and PCR detection

A total of 40 phage isolates (Table 1) were genetically characterized as part of this study. These isolates represent a subset of a larger phage collection of 120 phages isolated from whey samples which were obtained from dairy processing plants in various geographical locations from different continents (including Europe, North-America and Asia) and at various time points (being collected in the period 2006–2012, inclusive). Plaque assays were performed on suitable host strains, and single plaques were propagated as described in the Materials and Methods section. The resulting high titre, purified phage preparations were then employed in a phage-host survey, using a total of 91 potential host strains, in order to determine their host ranges. In the majority of cases, isolated phages infected their primary (i.e., the host on which they were originally propagated) host and one further strain—with a small number of phages infecting between three and six *S. thermophilus* strains—thus overall revealing a narrow host range (data not shown), which is consistent with previous observations for phages infecting this species (Binetti et al., 2005; Zinno et al., 2010). Following this analysis, and in order to assess the distribution of phage groups in the collection, phage isolates were subjected to multiplex polymerase chain reaction (PCR) grouping using the primer sets described by Quiberoni et al. (2006), which are designed to detect *cos*- and *pac*-site containing phage groups. While the vast majority of phages could be grouped using this method, eight isolates did not yield an amplicon using this PCR system employing either fresh lysate or extracted DNA as template material (discussed further below).

Three main criteria were used to select phages for sequencing: (i) phages which did not return a PCR product using the above mentioned phage grouping PCR and which were therefore considered novel, (ii) persistence and/or prevalence (as determined by similar phages being isolated persistently from the same dairy factories, or phages infecting two or more distinct *S. thermophilus* strains), and (iii) diversity (as determined by host range and restriction profiling of phage genomic DNA). For the purpose of this study, the results (and discussion thereof) below pertain to the subset of 40 phages whose genomes were sequenced, unless otherwise indicated.

As indicated in Table 1, *cos*-containing phages were the most frequently detected phage group (both in the 120-phage collection as a whole, as well as within the selected 40 phages), a dominance also observed—though to varying degrees—in previously analyzed phage collections (Zago et al., 2003; Guglielmotti et al., 2009a; Zinno et al., 2010). The reason for the observed dominance of *cos*-containing phages is not known.

**Figure 1:**
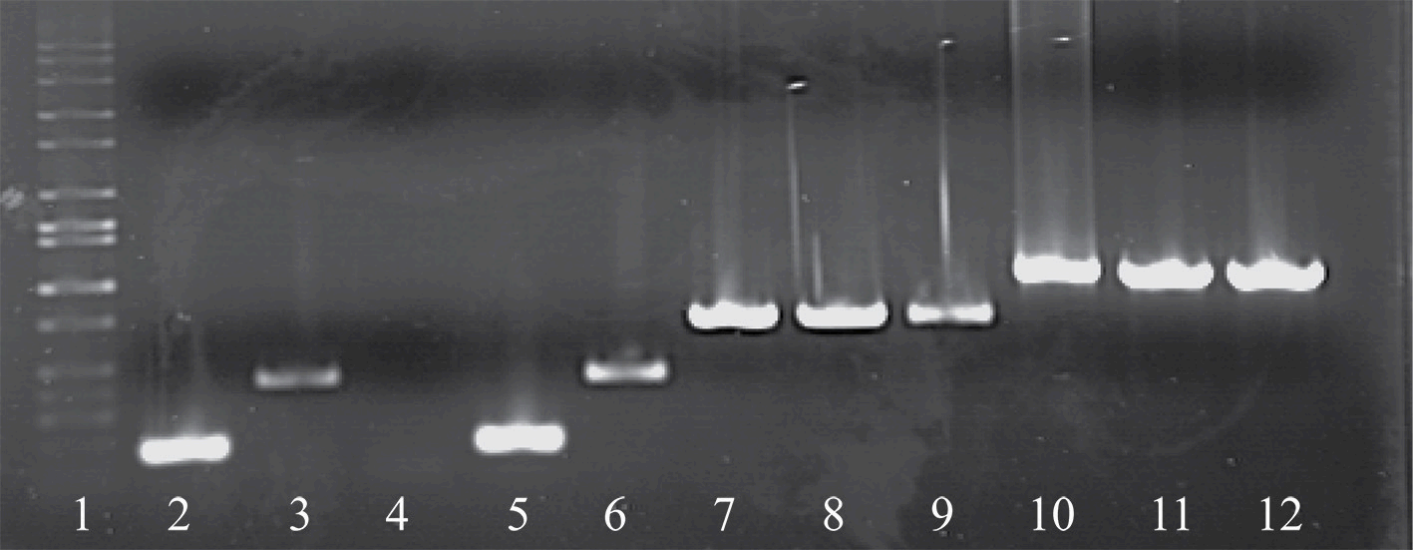
Electrophoretic examination of PCR products amplified from phage DNA (as template) using the multiplex PCR phage detection primers described in Table 2. Lane 1: 1 kb Full scale DNA Ladder (Fisher Scientific), Lane 2: 7201 (*cos*-containing control), Lane 3: O1205 (*pac*-containing control), Lane 4: negative control (sd H_2_O), Lane 5: 9851 (*cos-*containing), Lane 6: 9853 (*pac*-containing), Lanes 7-9: 987 group phages 9871, 9872, and 9873, Lanes 10-12: 5093 group phages 0093, 0094, and 0095.

As noted above, a total of eight phage isolates did not yield a PCR product using the standard *cos*- and *pac*-containing phage detection PCR. Genome sequencing was performed on these eight isolates, which revealed that four of these represent a novel group, designated the 987 group (these findings have been published elsewhere; McDonnell et al., 2016), with the remainder belonging to the 5093 group (and described below). These genome sequences were utilized to design a multiplex PCR primer set (Table 2), which allows detection of representatives of all four *S. thermophilus*-infecting phage groups (Figure 1, Lanes 5–12). The two newly designed primer sets were successfully tested on all four members of both the 5093 and 987 phage groups isolated in this study. Furthermore, considering that the primers used to detect the 987 (targeting Open Reading Frame 6 [*ORF6*] of phage 9871, which encodes a conserved scaffolding protein) and 5093 (targeting *ORF3* of phage 0095, which encodes a portal protein) phage groups produce products of different sizes (707 base pairs [bp] and 983 bp, respectively), these newly designed primer sets are fully compatible with each other and with the *cos* and *pac* phage-detecting pairs (Quiberoni et al., 2006). This multiplex PCR method thus embodies a very useful updated tool to detect representatives of all currently known phage groups infecting this starter species.

### Morphological analysis

**Figure 2:**
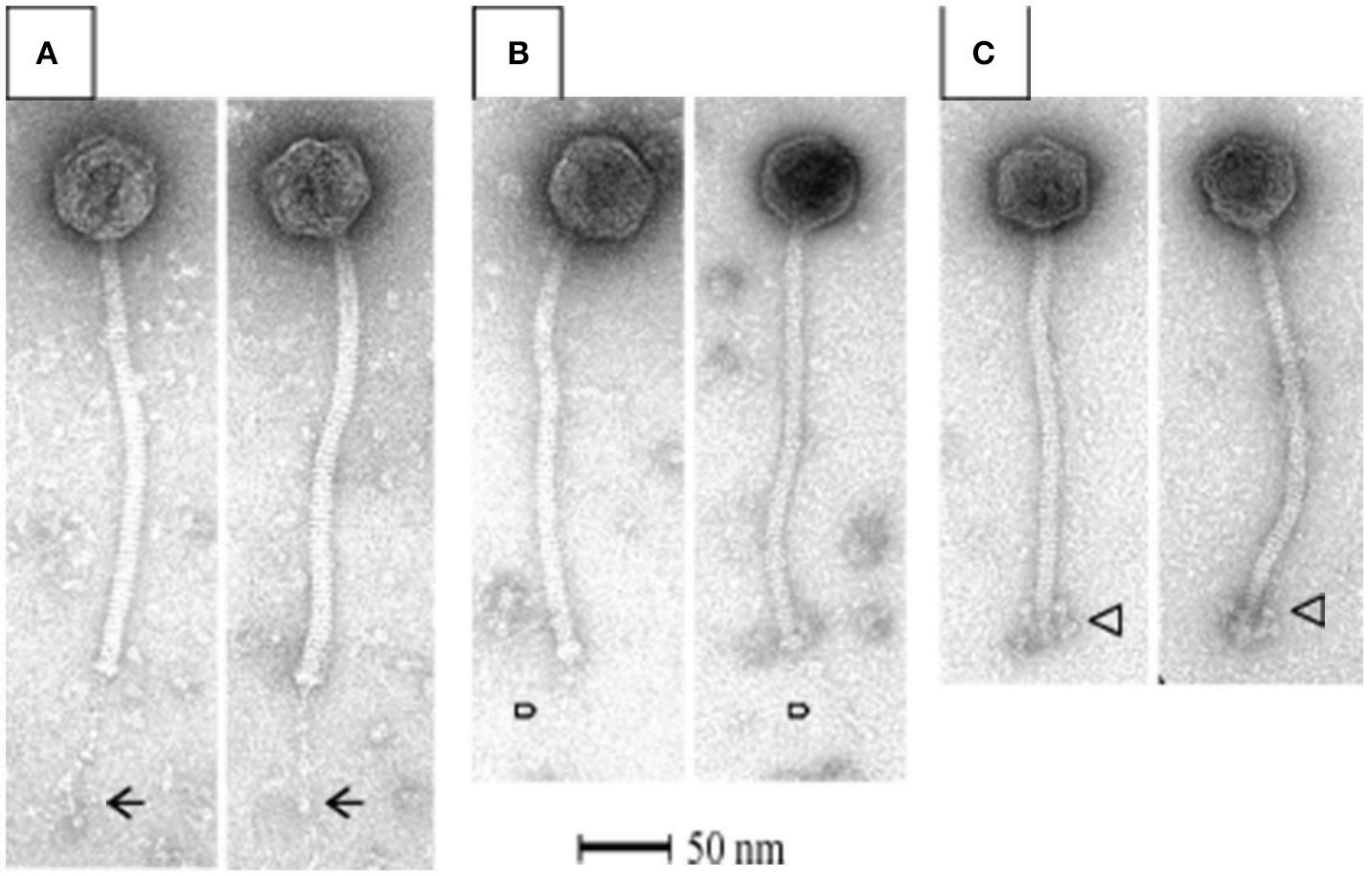
Electron microscopic analysis of representative *cos*-containing (phage 7573; **A**), *pac*-containing (7951; **B**) and 5093 group (phage 0092; **C**) phages visualized using uranyl acetate staining.

All *S. thermophilus* phages identified to date are classified as *Siphoviridae*, corresponding to type B as described by Bradley (1967), and thus possess icosahedral heads and non-contractile tails. Representative members of the phage collection employed in this study were selected for electron microscopic (EM) analysis, with members of the *cos*-containing, *pac*-containing and 5093 groups shown in Figures 2A–C, respectively), while EM analysis of members of the 987 group has been presented elsewhere (McDonnell et al., 2016). All analyzed phages display the expected morphology, particularly in terms of tail length, possessing tails that are longer than those of (the majority of) their lactococcal counterparts, i.e., 936, c2 and many P335 phages (Pedersen et al., 2000). Phages 7573 and 7951 exhibit an extended, thin tail fiber (Figures 2A,B; indicated by arrows), although genome analysis failed to identify candidate genes which may encode these interesting structures. Phage 0092 is a member of the 5093 group, whose morphology differs from the *cos*- and *pac*-containing group phages in that the tail tips are characterized by globular features that protrude from the base of the tail (Figure 2C; indicated by an arrow).

### Phage genome features

**Figure 3:**
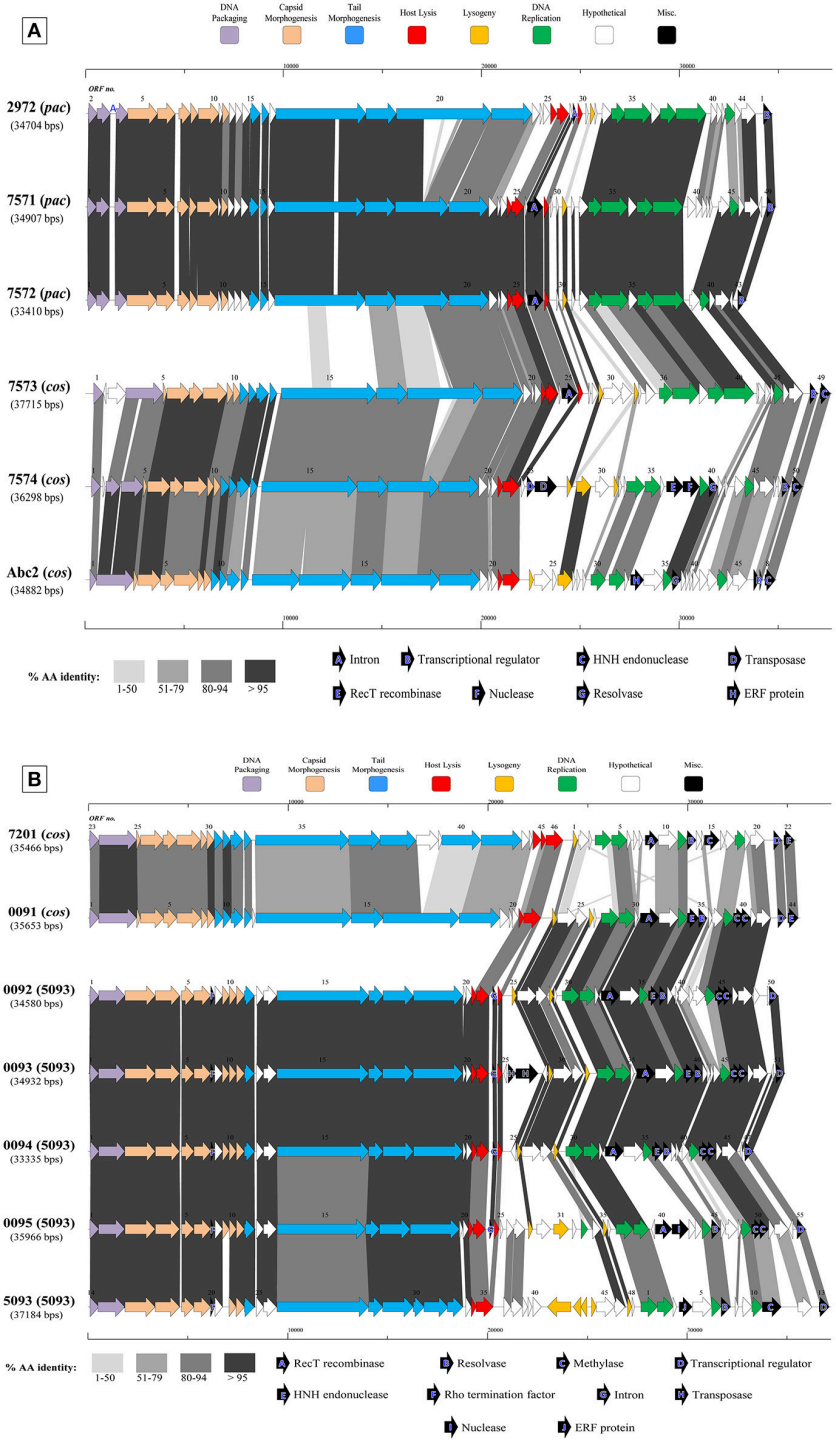
Comparative analysis of the genetic organization and content of sequenced phage genomes. **(A)** Phages infecting *S. thermophilus* strain ST68757. **(B)** Phages infecting *S. thermophilus* strain ST67009. Predicted ORFs (indicated by arrows) and gene products (putative function indicated by color coding) are aligned with adjacent genomes according to % amino acid identity (indicated by shaded boxes). Gene products considered to be notable are marked in black, with accompanying legend. See Table 1 for details on phages. Phages sequenced as part of this study are compared to previously sequenced *cos*-containing phages 7201 and Abc2, *pac*-containing phage 2972, and 5093 type phage 5093 for illustrative purposes.

The general characteristics of the sequenced phage genomes are given in Table 1, with predicted ORF schematics of representative phages displayed in Figure 3. An overall comparative analysis of all 40 genomes (based on percent nucleotide identity as well as divergence score) is provided in Supplementary Figure S1. The complete nucleotide sequences of a total of 26 *cos*-containing phages, 10 *pac*-containing phage and four 5093 group phages were determined, with the genomes of the four 987 group phages presented elsewhere (McDonnell et al., 2016). Genomes ranged in size from 33.3 to 38.1 kilobasepairs (kb) (the longest and shortest genomes sequenced are highlighted in bold in Table 1). The number of predicted ORFs in each genome ranged from 42 to 55 (increasing ORF number generally corresponding with increasing genome size), with GC content averaging 38.95%—consistent with the GC% content of previously published *S. thermophilus* genomes (Bolotin et al., 2004; Treu et al., 2014; Wu et al., 2014; Labrie et al., 2015). The 40 sequenced phage genomes are divided into three groups, based on amino acid identity of predicted ORF products to those of previously defined *cos*-containing, *pac*-containing and 5093 group phages (Le Marrec et al., 1997; Mills et al., 2011), which is in agreement with the results of the PCR-based typing system (discussed above).

The overall genomic organization, and amino acid conservation within defined groups, of the sequenced phages is comparable with that of previously published examples (Levesque et al., 2005; Guglielmotti et al., 2009b; Mills et al., 2011), indicating that this modular arrangement is highly conserved amongst these phages, even between distinct phage groups across a large number of samples. The structural protein-encoding genes of representatives of these phage groups have been defined previously (Levesque et al., 2005; Duplessis et al., 2006; Guglielmotti et al., 2009b; Mills et al., 2011; Szymczak et al., 2016), with the known structural protein-encoding genes in the 5093 group being expanded upon here. The functional annotation of the genes encoding tail components of *S. thermophilus* virions (specifically those encoding the distal tail (Dit), tail-associated lysin (TAL), antireceptor and baseplate components) was carefully performed in this study, in particular for members of the 5093 group, and also exhibit functional synteny between phage groups despite substantial nucleotide divergence (Figure 3; McDonnell et al., 2016).

Figure 3A details the predicted ORFs of five phages infecting *S. thermophilus* ST67009 (one *cos*-containing phage and four 5093 group phages), confirming the genetic conservation of members of the 5093 lineage. The amino acid divergence between proteins encoded by the structural regions of this and other groups, previously observed by Mills et al. (2011), is evident. This is despite the conservation of the 7201-like/Group II replication module (discussed below) in all five phages.

*Streptococcus thermophilus* phage replication modules are defined here as the genomic regions immediately following the lysogeny replacement module (i.e., that region containing presumably remnant genes involved in the phage lysogenic cycle; Lucchini et al., 1999), and preceding the gene encoding the small subunit of the terminase. These regions contain genes encoding predicted primosome components, DnaC-like proteins (helicases), single-stranded DNA and DNA-binding proteins, endodeoxyribonucleases related to RusA, primases and replisome organizers, amongst various genes with hypothetical functions. Akin to the structural modules, *S. thermophilus* phage replication modules can be divided into (at least) two distinct groups, namely the Sfi21- and 7201-like, or Group I and II (Stanley et al., 2000; Brussow and Desiere, 2001); the former being detected most frequently in phages in the present study (present in 27 out of 40 examined phages), which is consistent with previous studies (Desiere et al., 1997; Stanley et al., 2000; Brussow and Desiere, 2001). The replication modules of two phages (7951 and 7955; Table 1) do not appear to fall into either of the two above mentioned categories (based on BLAST searches), and therefore may be included in the “non-I/non-II” grouping as previously proposed by Stanley et al. (2000). In contrast with other features of these phages, such as structural protein content (Le Marrec et al., 1997), there appears to be no correlation between possession of the Sfi21- or 7201-like replication modules and mode of DNA packaging, e.g., in the cases of phages 7573 and 7574 (Figure 3B). This phenomenon may be due to modular rearrangements (Lucchini et al., 1999) producing a seemingly random distribution of discrete genomic segments in *S. thermophilus* phages. The “rightward” genomic region (defined here as the region at the 3′ end of the lysis module) is often characterized by insertions, deletions, and point mutations leading to a large degree of heterogeneity (Lucchini et al., 1999; Figure 3). Indeed, this was found to be the case in these segments of the phage genomes analyzed in the present study, which were (in some cases) observed to encode gene products of apparently non-streptococcal phage origin (e.g., RecT recombinases and transposases, indicated in the legends of Figures 3A,B).

Interestingly, several proteins predicted to be involved in (mostly host) DNA recombination are encoded by 12 phages in the present collection-either singly or in combination (Table 1). Among those identified are RecT and ERF family proteins (Hall and Kolodner, 1994; Noirot and Kolodner, 1998; Passy et al., 1999). These protein superfamilies have previously been detected in phages of low GC, Gram-positive bacteria, including *S. thermophilus* phage 7201 (Stanley et al., 2000) and *L. lactis* phage r1t (van Sinderen et al., 1996), and are generally associated with the presence of endodeoxyribonucleases of the RusA family (Sharples et al., 1994; Macmaster et al., 2006), as well as MTases and single-stranded DNA-binding proteins (Iyer et al., 2002), as is the case in a number of phages sequenced in this study (Table 1). In a similar fashion, recently described bacterial RecT-encoding genes have been associated with genes encoding putative exonuclease genes, and this “modular” combination was also observed on the genomes of four phages sequenced in the present study (Datta et al., 2008; phages 5641, 7574, 7951, and 7955; Table 1).

The presence of HNH endonuclease-encoding genes in a 3′ “terminal” position (i.e., present downstream of predicted transcriptional regulator-encoding genes but preceding the deduced *cos*-sites) was found to correlate with mode of DNA packaging in each phage. Those phages utilizing the *cos* mode of DNA packaging possessed a terminal HNH endonuclease, whereas those predicted to utilize the *pac* mode did not (Table 1). This finding is consistent with previous studies examining the role of HNH endonucleases in the DNA packaging process, and specifically in *cos*-site cleavage (Kala et al., 2014).

### Panvirome analysis

**Figure 4:**
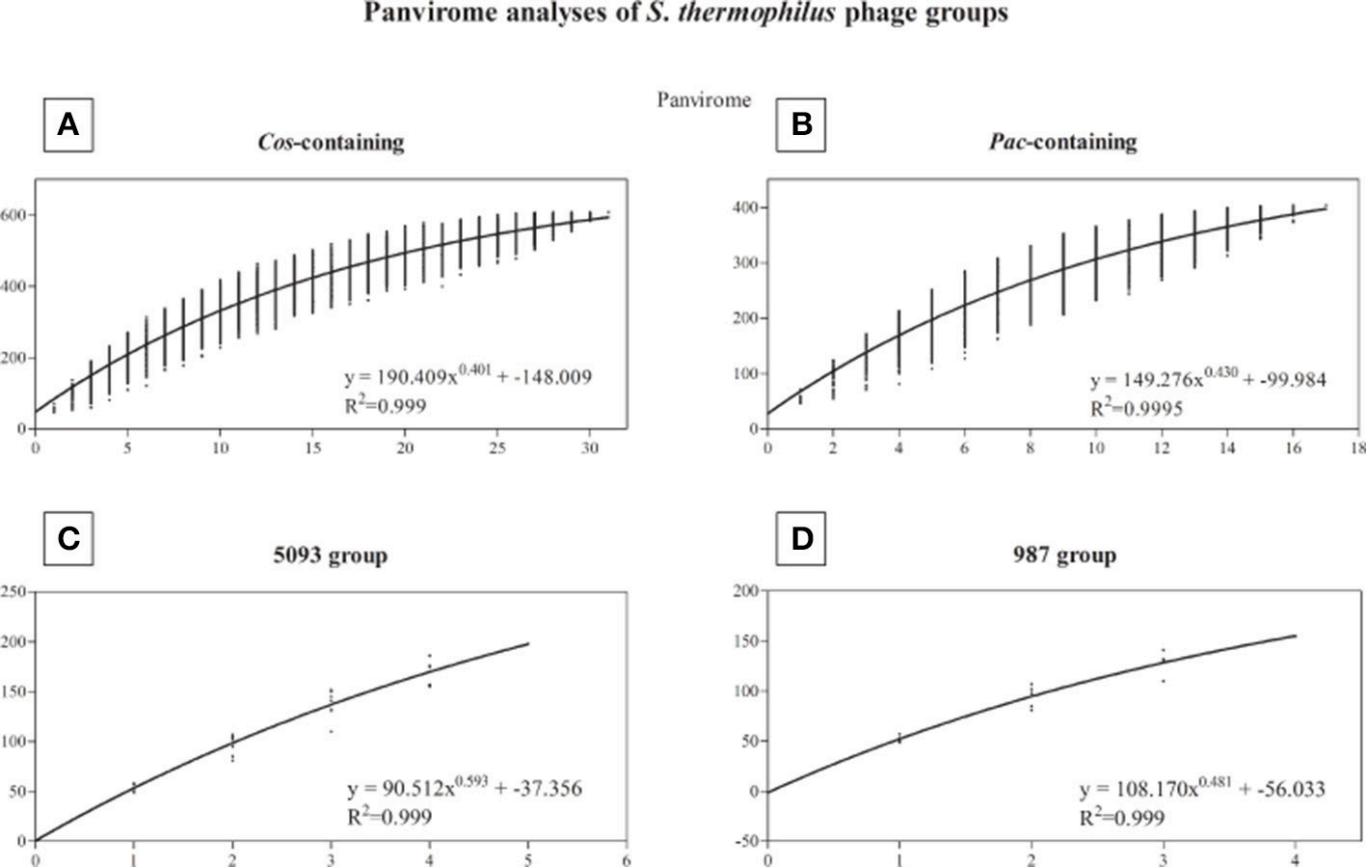
Panvirome analysis of the four currently known groups of phages infecting *S. thermophilus*. **(A)** *cos*-containing group (*n* = 31), **(B)** *pac*-containing group (*n* = 17), **(C)** 5093 group (*n* = 5), **(D)** 987 group (*n* = 4). Exponent and *R*^2^ values are given in the inset of each graph.

A pan-genome analysis of all phages (“pan-virome”) sequenced in this study, the 987 group phages as well as 13 previously sequenced (and publically available) *S. thermophilus* phage genomes was conducted as described in the Methods section, the results of which are shown in Figure 4. The exponential values of each analysis (Figures 4A–D, inset) indicate that, at least in the case of the *cos*-containing, *pac*-containing and 987 groups, sequencing of additional genomes of phages in these groups may not lead to a significant increase in known genetic diversity. However, due to the low number of complete genomes available for the 987 group phages (a total of 4 analyzed in this study), this conclusion cannot be drawn, despite the exponent value of <0.5 (Figure 4D). The inclusion of further genome sequences, such as those of the recently published P335-like *S. thermophilus* phages (Szymczak et al., 2016), in future studies, may be pertinent in order to overcome this limitation. An exponent value of >0.5 which was calculated for the 5093 group phages indicates an “open” pan genome and highlights the usefulness of whole genome sequencing of additional members of this novel group. In general, and as discussed above, conservation was observed within phage groups across the structural regions of the genomes, while the lysis and replication modules displayed an increased genetic divergence. In addition, no gene was identified with homologs across every individual member of the four phage groups (core genome = 0), indicating the high level of genetic diversity present in phages infecting *S. thermophilus*.

### Notable genomic features

#### Methyltransferases

The process of self-methylation of genomic DNA has been acknowledged as a protective strategy employed by phages against host-encoded restriction-modification (R/M) systems (reviewed by Murphy et al., 2013), which have been detected previously in *S. thermophilus* (Burrus et al., 2001; Goh et al., 2011; Labrie et al., 2015). In total, 12 predicted MTase-encoding genes were detected on 7 phage genomes sequenced as part of this study. In order to prove that phage-encoded MTases protect such *S. thermophilus* phages against DNA restriction, one deoxyadenosine (DAM) MTase-positive phage (indicated by the similarity of the protein product of this MTase to GATC-specific methytransferases, observed using the REBASE tool; Roberts et al., 2015), and one MTase-negative phage infecting the same strain (as control) were subjected to restriction profile analysis using DpnI and DpnII restriction endonucleases, as described previously (Murphy et al., 2014). It is known that DpnI only targets DNA which is DAM-methylated, while DAM-methylation protects DNA from restriction by DpnII (Lacks and Greenberg, 1977). Supplementary Figure S2 clearly shows that while the genome of phage 9901 (MTase-negative) is restricted by DpnII (and not by DpnI; Lanes 2 and 3), the genome of 9902 (MTase-positive) was restricted by DpnI (and not by DpnII; Lanes 4 and 5), thus validating the protective effect and functional activity of this MTase.

Predicted MTase-encoding genes were also present on the genomes of each of the four 5093 group phages {0092 to 0095 (and, interestingly, also on 5093; Mills et al., 2011)}, although these were of a different type than those discussed above, and predicted (using a BLAST search; Altschul et al., 1990) to represent cytosine-5 methyltransferases (C5 MTase; reviewed by Kumar et al., 1994). A striking feature of these particular MTases is their apparent total overlap, being produced as translation products coded by the same DNA in the same position on the phage genome, but in different reading frames (Figure 3A, indicated by “C”). One of these products bears amino acid similarity to a C5 MTase present in the temperate *Streptococcus pneumoniae* phage (Obregon et al., 2003), where a similar overlap was identified as previously also found in a conjugative streptococcal transposon (Sampath and Vijayakumar, 1998). The putative origin of these genes is consistent with the amino acid similarity of many gene products in the 5093 group phages to non-dairy streptococcal phages, a connection which has been explored previously in prophages of pathogenic streptococci (Desiere et al., 2001).

#### Introns

Introns which interrupt lysin-encoding genes are known to be widely distributed in *S. thermophilus* phages (Foley et al., 2000; Ali et al., 2014). A number of putative introns were indeed detected on the genomes of phages in the current study, though their function is unknown. In total, 19 group IA introns were identified in lysis modules, based on an interrupted lysin-encoding gene and/or the presence (with varying nucleotide identity) of a 14 bp consensus sequence correlated with the possession of an intron (Foley et al., 2000).

#### Transcriptional regulation

Late transcriptional regulators (Ltr's) in phages infecting Gram-positive bacteria have been implicated in DNA packaging and lysis (Quiles-Puchalt et al., 2013), and a role for Ltr encoded by certain *S. thermophilus* phages has previously been proposed by Ventura et al. (2002). Ali et al. (2014) observed an ArpU-like transcriptional regulator-encoding gene preceding the gene specifying the small subunit of the terminase in temperate phages TP-J34 and TP-778L, similar in organization to the Ltr-encoding gene which was recently identified as a member of a superfamily of Ltr's present in phages of Gram-positive bacteria (Quiles-Puchalt et al., 2013). The genomic position of these genes is similar to the gene encoding a late transcriptional regulator identified in *L. lactis* phage TP901-1 (Brondsted et al., 2001). These findings prompted us to conduct an *in silico* analysis of sequenced *S. thermophilus* phages to determine the prevalence of Ltr homologs and functional equivalents in phages of this species. All previously sequenced *S. thermophilus* phages (including the recently described 987 group phages), as well as those sequenced as part of this study were observed to encode an Ltr-like protein, which either belongs to the ArpU family or the Ltrb family (DUF1492; Quiles-Puchalt et al., 2013). With the exception of phage ALQ13.2 (Guglielmotti et al., 2009b) and 4761 (this study), the possession of a regulator of the Ltrb family correlates with phages utilizing the *cos* mode of DNA packaging, and possession of ArpU with the *pac* mode. This correlation is consistent with the role of the Ltr protein families in regulating the expression of the genes encoding DNA packaging machinery in other phages (Quiles-Puchalt et al., 2013).

### Phage VR2 sequence clustering

The antireceptor-encoding genes of the phages sequenced in this study were generally conserved (with the exception of the 5093 group phages, whose antireceptor-encoding genes are discussed separately below), and found (using the NCBI CDD tool; Marchler-Bauer et al., 2015) to contain distinct domains, separated in some cases by collagen-like repeats as described previously (Duplessis and Moineau, 2001). *S. thermophilus* phage antireceptor proteins have previously been shown to harbor variable regions, one of which (VR2) is purported to be the main host range determinant (Duplessis and Moineau, 2001). An unrooted relatedness tree showing the genetic distance between the deduced amino acid sequences of these VR2 regions in the present phage collection is shown in Supplementary Figure S3 and in which VR2 regions are color-coded by host. In the majority of cases, the VR2 regions cluster according to host strain, but is not phage group-dependent, i.e., the VR2 regions of *cos*- and *pac*-containing phages are not aligned in the same cluster (as has similarly been observed; Binetti et al., 2005). The presence of outliers is not surprising, as it has been suggested that the VR2 region may not be the only host determinant operating in *S. thermophilus* phages (Duplessis et al., 2006). A comprehensive analysis of the multiple variable regions in the tail gene-encoding modules of *S. thermophilus* phages, as well as sequence and structural information on host-encoded receptors, may be required to reconcile the observed VR2 region anomaly.

### 5093 phage group structural protein identification

**Figure 5:**
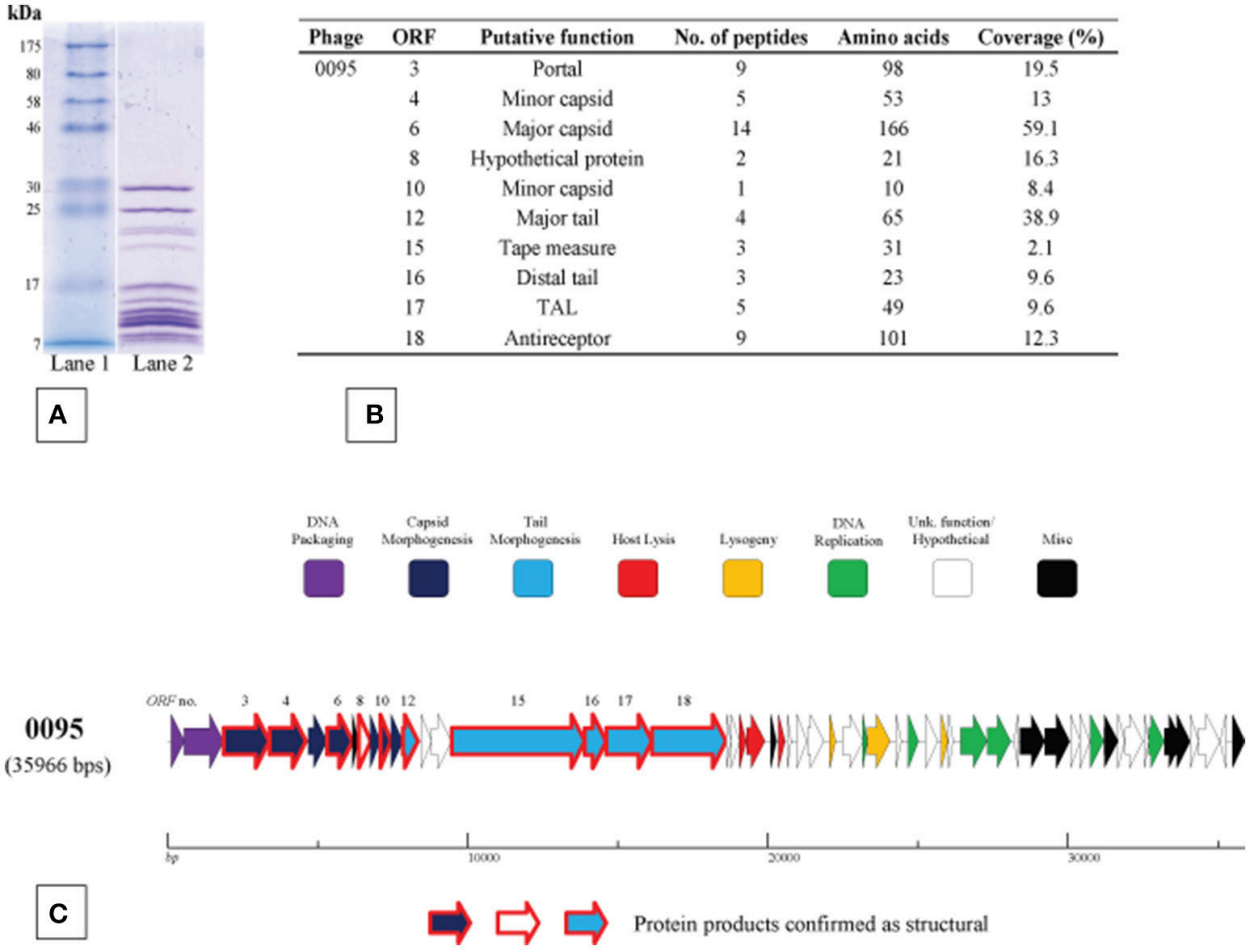
Structural proteome analysis of phage 0095. **(A)** SDS-PAGE gel (12%) showing the structural protein profile of phage 0095. Lane 1: Broad range protein ladder (New England Biolabs); Lane 2: phage 0095 protein extraction. **(B)** Deduced structural proteins (and corresponding ORF number) as identified by ESI-MS/MS (threshold: two unique peptides or 5% ORF coverage). **(C)** Predicted ORF schematic of phage 0095 highlighting confirmed structural protein-encoding genes (bold outline in red).

The structural protein complement of phage 0095, as a representative of the 5093 phage group, was determined by mass spectrometry (Figure 5) as described in the Materials and Methods section. Three protein products hypothesized to form the phage tail tip and so-called “initiator complex” (Dit, TAL and antireceptor) were all detected as structural components of the 0095 virion. As expected, the presumed major capsid protein and major tail protein were also detected (Figure 5), of which homologs had previously been identified for phage 5093 (Mills et al., 2011) and CHPC1151 (Szymczak et al., 2016). Furthermore, the predicted portal protein, the putative tape measure protein (TMP), and several minor structural proteins were identified (Figure 5), significantly expanding the experimentally-determined identification of structural proteins for this phage group. The confirmation of the structural nature of these protein products, as well as the genomic position (and, indeed, order) of the corresponding genes confirms an overall functional synteny in this region across all four *S. thermophilus*-infecting phage groups (namely the *cos*-containing, *pac*-containing, 5093 and 987 groups).

### Identification of the 5093 phage group antireceptor

The 5093 genome appears to lack an assigned antireceptor-encoding gene (Mills et al., 2011). Phages are expected to encode an antireceptor as such proteins are responsible for the initial binding of the phage to the bacterial cell surface thereby determining host specificity (Duplessis and Moineau, 2001). Assuming that the conserved modular genomic structure of *S. thermophilus* phages (discussed above) also applies to phage 5093, *ORF29*_5093_ is predicted to encode the TMP, known to be responsible for phage tail length (Pedersen et al., 2000). This is borne out by its large size (the encoded protein is approximately 1500 amino acids in length) and the presence of extensive helical structures, as predicted by the PSIPRED tool (Jones, 1999). Downstream of *ORF29*_5093_, *ORF30*_5093_ is presumed to specify the distal tail protein (Dit), based on a HHPred-mediated match for this product to the Dit protein of *Bacillus* phage SPP1 (*P* = 6.2 × 10^−31^). The product of the next gene (*ORF31*_5093_) shows similarity to phage endopeptidases using BLAST and Pfam searches, and its position on the genome is consistent with a common lactococcal phage tail structure, namely the tail-associated lysin or TAL (Kenny et al., 2004; Mc Grath et al., 2006; Stockdale et al., 2013).

*ORF32*_5093_, *ORF33*_5093_ and *ORF34*_5093_ are predicted to encode two hypothetical proteins and a hydrolase, respectively. However, in the four 5093 group phages sequenced in the current study, these three deduced ORFs form instead a single ORF (for example *ORF18*_0095_ in the case of phage 0095; Figure 3A), the product of which is 821 amino acids in length. We propose that this single ORF encodes the antireceptor (henceforth termed RBP, including a subscript number to indicate the particular phage it is derived from, e.g., RBP_0095_) in this group of phages (Figure 3A). This hypothesis is based on: (i) the gene position at the 3′ end of the tail morphogenesis module, but preceding the lysis module, consistent with typical RBP-encoding gene positions (Duplessis and Moineau, 2001; Mahony et al., 2013; Casey et al., 2014) and (ii) the presence, at the C-terminal end of the protein, of a GDSL-family esterase domain, detected using Pfam, CDD, and HHPred. GDSL lipases are members of the diverse SGNH hydrolase superfamily, and examples include the Axe2 acetylxylan esterase, responsible for the cleavage of acetyl side chains from xylooligosaccharides, such as xylan (Lansky et al., 2014). These similarities suggest a carbohydrate binding function for this protein product. Taken together, and further corroborated below using an adsorption inhibition assay, these findings lead us to assign the antireceptor function to this gene product. It is not known if phage 5093 possesses several truncated forms of this particular ORF or if the observed differences are due to sequencing errors.

**Figure 6:**
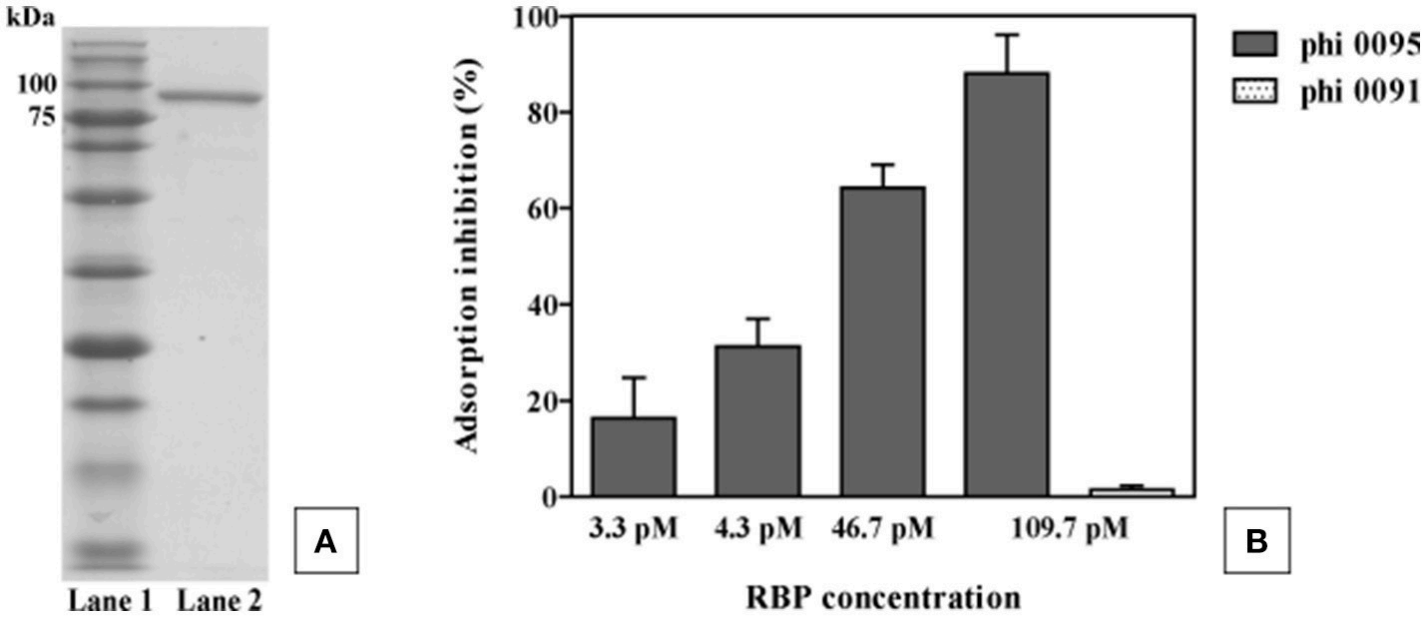
Phage 0095 adsorption inhibition analysis using varying concentrations of purified RBP_0095_ on *S. thermophilus* ST67009 by blocking assay. **(A)** SDS-PAGE gel (12%) showing purified antireceptor of phage 0095. Lane 1: Blue prestained protein standard, Broad range (New England Biolabs), Lane 2: purified 0095 antireceptor. **(B)** % inhibition of 0095 adsorption on ST67009. Phage 0091 is included as an adsorption inhibition-negative control using 109.7 pM RBP_0095_.

To verify its role as the antireceptor, RBP_0095_ was heterologously expressed and purified (Figure 6A), resulting in a protein with a molecular weight of approximately 90 kilodaltons (kDa), consistent with the calculated molecular weight (of 92.1 kDa). Figure 6B clearly shows that this purified antireceptor, when incubated with ST67009 cells (host for phage 0095) at varying concentrations, inhibits adsorption of phage 0095 to the host in a dose-dependent manner. Phage 0091 (a *cos*-containing phage also infecting ST67009) was included as a negative control to indicate the specificity of RBP_0095_ in inhibiting the adsorption of phage 0095 (but not that of phage 0091; Figure 6B). The concentrations observed to exert blocking of phage adsorption were comparable to those previously observed using the 987 group antireceptor (McDonnell et al., 2016) and that of lactococcal phages Tuc2009 and TP901-1 (Collins et al., 2013). In the present study, specific concentrations of RBP_0095_ (Figure 6B) were selected to illustrate an even distribution of adsorption blocking levels. This dose-dependent reaction indicates that RBP_0095_ is indeed responsible for host interaction of this phage, which may be extended to the other ST67009-infecting 5093 group phages based on the high level of amino acid identity (99%) between the gene products (Figure 3A). Considering the lytic nature of these phages, and the fact that they appear to be emerging in commercial fermentations (having been either absent or undetected until 2011), this information may be industrially valuable, and the above data represent a significant step forward in the annotation and characterisation of these recently discovered (and potentially important) phages.

## Conclusions

The genomes of phages infecting *S. thermophilus*, which are a recurring problem in the dairy industry, are conserved in terms of modular genomic arrangements, but divergent between defined *cos*-containing, *pac*-containing, 987 and 5093 groupings. Complete genome sequencing has enabled the development of a multiplex detection PCR capable of rapidly identifying individual members of these four phage groups, and shed light on their mechanisms of proliferation, their adaptation to their environment and to host-encoded defenses. These include - but are not limited to - genome modular rearrangements, point mutations and phage-encoded methyltransferases. Structural protein identification through mass spectrometry and antireceptor definition by means of an adsorption inhibition assay were used here to characterize a representative member of the recently discovered 5093 group, substantially improving the annotation of members of this group.

To our knowledge this study represents the largest single (published) undertaking of *S. thermophilus* phage whole genome sequencing. The increased database of phage genomes provides a valuable knowledge resource to the dairy industry which relies on up-to-date phage detection and phage-host interaction information in order to successfully implement rotational schemes which minimize the economic impact of phage fermentation contamination.

## Declaration

The datasets generated and/or analyzed during the current study are available in the GenBank repository, https://www.ncbi.nlm.nih.gov/genbank/, under accession numbers KY705251-KY705290, inclusive.

## Author contributions

BM was involved in the experimental design and work and prepared the manuscript. JM was involved in the experimental design and work and data analysis interpretation. LH performed the initial processing of whey samples and isolation of phages. HN performed the electron microscopy. JN performed the mass spectrometry. GL and MV performed the Illumina sequencing of phage genomes. TK was involved in the experimental design and manuscript editing. DS was involved in the experimental design, data interpretation and manuscript editing and preparation. All authors read and approved the manuscript.

### Conflict of interest statement

A patent application describing elements of this work has been submitted. BM is funded by DSM Food Specialties, and LH and TK are employees of DSM Food Specialties. The other authors declare that the research was conducted in the absence of any commercial or financial relationships that could be construed as a potential conflict of interest.

## Abbreviations

CRISPR: clustered regularly interspaced short palindromic repeats
DAM: deoxyadenosine
MTase: methyltransferase-encoding gene
PCR: polymerase chain reaction
BIM: bacteriophage-insensitive mutant
bp: base pair
ORF: open reading frame
Dit: distal tail
TAL: tail-associated lysin
R/M: restriction-modification
Ltr: late transcriptional regulator
TMP: tape measure protein
RBP: receptor binding protein
kDa: kilodaltons
RSM: reconstituted skimmed milk.
