# Supplementary material for: Global Survey and Genome Exploration of Bacteriophages Infecting the Lactic Acid Bacterium Streptococcus thermophilus
Source: Front Microbiol. 2017 Sep 12;8:1754. doi: 10.3389/fmicb.2017.01754 (PMC5601072; doi:10.3389/fmicb.2017.01754)
Supplement: Supplementary file 1 [file DataSheet1.DOCX]

Supplementary Material

Global survey and genome exploration of bacteriophages infecting the lactic acid bacterium *Streptococcus* *thermophilus*

**Brian McDonnell, Jennifer Mahony, Laurens Hanemaaijer, Horst Neve, Jean-Paul Noben, Gabriele Andrea Lugli, Marco Ventura, Thijs Kouwen and Douwe van Sinderen^*^.**

*Corresponding Author: d.vansinderen@ucc.ie

## Supplementary Figures


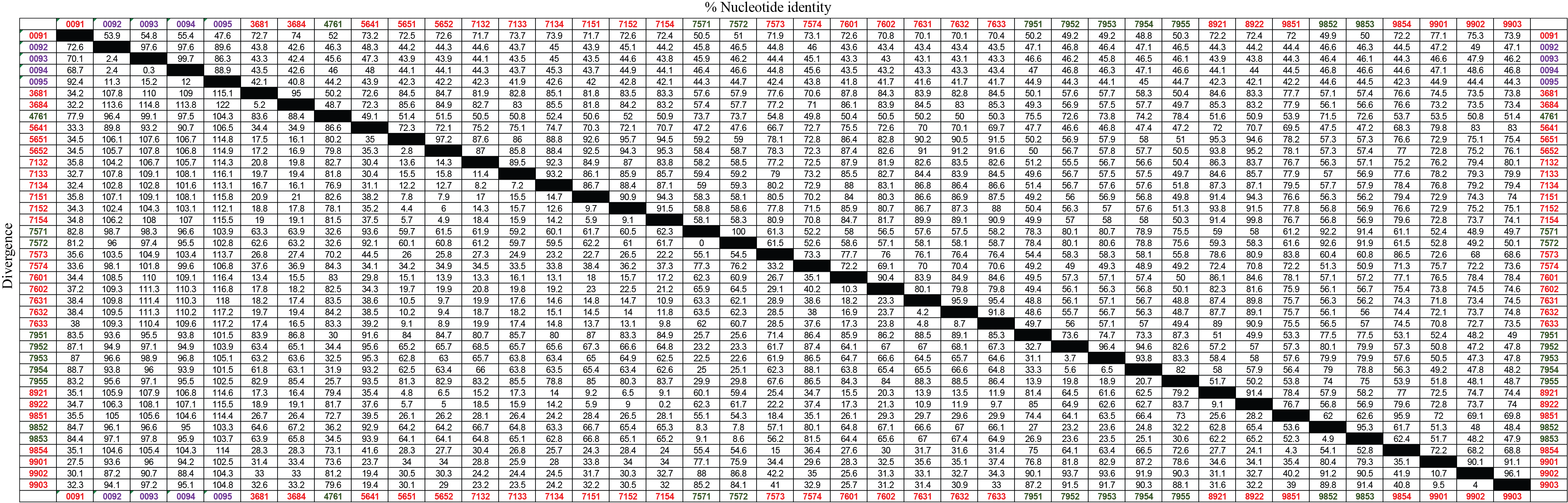


**Supplementary Figure S1.** Multiple alignment of 40 phage genomes, performed using Megalign (DNASTAR). Sequence distances are represented by (i) percent nucleotide identity (top right half) and by divergence score (bottom left half). Black squares represent the result of each genome aligned against itself, i.e. 100 % nucleotide identity or a divergence score of 0. *Cos*-containing phages are represented by red text, *pac*-containing phage by green text, and 5093 group phages by purple text.

**
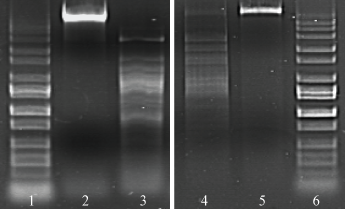
**

**Supplementary Figure S2.** Restriction profile analysis of MTase positive and negative phage genomic DNA. Lane 1, 6: 1 kb Full scale DNA Ladder (Fisher Scientific), Lane 2: 9901 (DpnI restricted), Lane 3: 9901 (DpnII restricted), Lane 4: 9902 (DpnI restricted), Lane 5: 9902 (DpnII restricted).


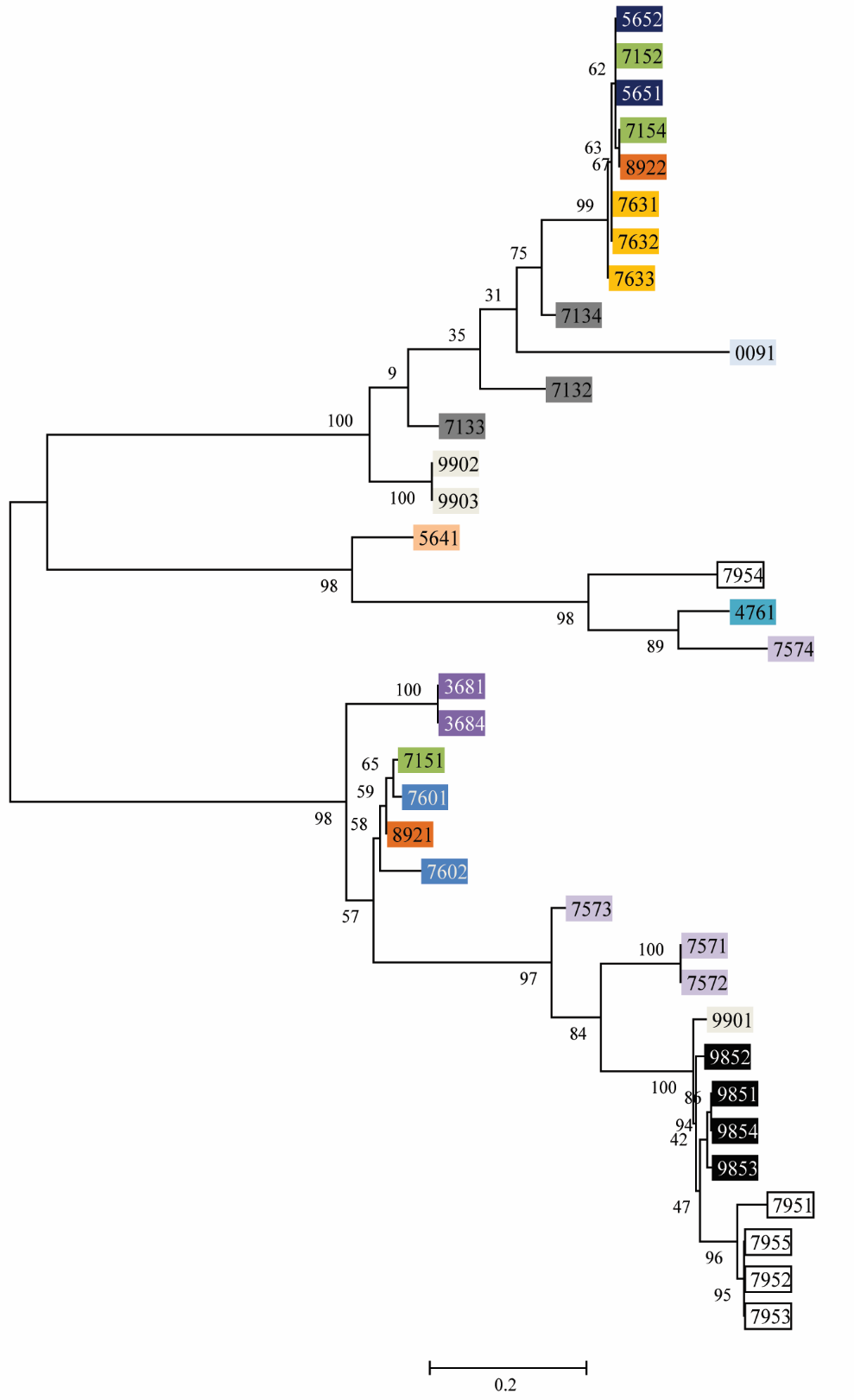


**Supplementary Figure S3.** Unrooted genetic relatedness tree showing the relationship between the VR2 regions of sequenced *pac*-containing (Black (9852, 9853), Blue (4761), Purple (7571, 7572), White (7951-7955) and *cos*-containing (all others) phage genomes. VR2 regions are colour-coded by host strain of the phage from which the analysed sequence was derived.
